# Prevalence of and Factors Associated With Eustachian Tube Dysfunction Among the Public in Jeddah, Saudi Arabia: Cross-Sectional Survey-Based Study

**DOI:** 10.2196/14640

**Published:** 2020-11-19

**Authors:** Khalid A Alshehri, Omar M Saggaf, Hussein M Alshamrani, Abdulrahman Mutlaq Alnefaie, Khalid B Alghamdi

**Affiliations:** 1 Otorhinolaryngology and Head and Neck Surgery King Abdulaziz University Jeddah Saudi Arabia; 2 Otorhinolaryngology and Head and Neck Surgery Prince Mansour Military Hospital Taif Saudi Arabia

**Keywords:** Eustachian tube, Eustachian tube dysfunction, chronic otitis media, hearing loss, electronic survey, cholesteatoma, 7-item Eustachian Tube Dysfunction Questionnaire

## Abstract

**Background:**

Obstruction of the Eustachian tube is a common condition that is unpleasant and might lead to various middle ear disorders.

**Objective:**

This study aimed to estimate the prevalence of Eustachian tube dysfunction (ETD) among the public in Jeddah, Saudi Arabia.

**Methods:**

This cross-sectional survey-based study was conducted in Jeddah during August 2018 by distributing an electronic survey form to participants from different districts of the city. All male and female residents of Jeddah aged 10 years and above had the chance to participate in this study.

**Results:**

A total of 2372 participants (female, 1535/2372, 64.71%; male, 837/2372, 35.28%; mean age 31.31 years, SD 11.85 years) agreed to contribute to our study. Upon analysis of their answers to the questionnaire, the overall prevalence of ETD in our sample was found to be 42.49% (1008/2372). The prevalence was higher among participants who reported a previous diagnosis of ETD and hearing loss (1897/2372, 80.00% and 1902/2372, 80.21%, respectively). Additionally, participants with a family history of hearing loss had a significantly higher prevalence (1136/2372, 47.92%) of ETD than those with no family history of hearing loss. Our analysis also showed that females were at a greater risk of developing ETD than males (*P*=.01).

**Conclusions:**

As per our prevalence data, ETD is a common disease in Jeddah, pointing to the need for more attention, awareness, and research.

## Introduction

The Eustachian tube is a thin tube that links the middle ear with the nasopharynx. It is responsible for ventilation and pressure equalization in the middle ear [1]. Therefore, any dysfunction of the Eustachian canal may lead to impaired sound conduction. Eustachian tube obstruction is a common condition that is unpleasant and might lead to various middle ear disorders such as chronic otitis media (OM) and cholesteatoma [2]. Eustachian tube dysfunction (ETD) can be due to intrinsic causes such as anatomical anomalies in the cleft palate, or acquired and extrinsic factors such as an allergic response at the site of the Eustachian tube opening, a viral upper respiratory tract infection, or a combination of intrinsic and extrinsic factors [3].

The prevalence of ETD among the adult general population is approximately 1% [4], and around 40% of children develop at least transient ETD [5]. Several studies have determined that ETD exists in up to 70% of patients undergoing tympanoplasty for middle ear disorders such as cholesteatoma and chronic OM [6,7]. A recent study conducted in the United States assessed the burden of ETD and determined that the condition is related to more than 2 million clinic visits per year among patients aged 20 years and above [8]. Some possible consequences of ETD include communication problems, decreased productivity, and poor quality of life. One of the main consequences of ETD is OM with effusion (OME), which is a common cause of hearing loss and is related to speech delay among children [9].

Patients with ETD complain of ear fullness, pain, muffled hearing, “popping” sounds, or tinnitus [6]. Hence, to diagnose ETD, a patient must exhibit the abovementioned symptoms of pressure disequilibrium in the involved ear. Nonetheless, it has always been challenging to identify appropriate diagnostic methods and tests as well as establish criteria for identifying individuals with ETD [6]. Several tools have been reported for evaluating Eustachian tube function. However, the use of Eustachian tube function tests is limited by the requirement of expensive tools and trained staff, which are commonly available in specialized health centers only [10]. Thus, a simple instrument such as a questionnaire that can reliably identify individuals with ETD would be a helpful tool for in-office practice, since it is the most appropriate way to self-evaluate a patient’s complaints and symptoms. The 7-item Eustachian Tube Dysfunction Questionnaire (ETDQ-7) is considered to be a disease-specific instrument for evaluation of the symptoms associated with obstructive ETD and its treatment outcomes [11].

Knowledge of the prevalence of a disease within a population is of value in establishing possible current and future community service requirements. To the best of our knowledge, no study in Saudi Arabia has assessed the prevalence of ETD in the community to date. This study aims to evaluate the prevalence of ETD in Jeddah, Saudi Arabia, by using the ETDQ-7 instrument.

## Methods

### Questionnaire

This study was approved by the institutional review board of King Abdulaziz University Hospital, Saudi Arabia. This cross-sectional survey-based study was conducted by distributing an electronic questionnaire among the general population of Jeddah during August 2018. The electronic questionnaire only accepted a one-time response per participant so that there would be no instances of multiple responses from the same person. Jeddah, an urban metropolis and one of the biggest cities in Saudi Arabia, is located in the western region of the country and has a population of approximately 3.5 million.

### Participants

This study aimed to estimate the prevalence of ETD in all areas of Jeddah. An electronic survey form was distributed by trained medical students to the participants. Randomly distributed city districts were selected for the study in order to obtain a realistic representative sample of the population of Jeddah, taking into account the more densely populated northern and southern regions of the city. All male and female residents of Jeddah aged 10 years and above had the chance to participate in this study. The purpose of the study was disclosed to the participants, and written consent was obtained before they could fill out the questionnaire.

### Measurements

The ETDQ-7, designed by McCoul et al [11] 5 years prior to this study, was established as a new scoring system for the evaluation of the symptoms associated with obstructive ETD [11]. Each of the 7 items have scores ranging from a minimum of 1 to a maximum of 7 points, resulting in a total score of between 7 and 49 points. A total score≥14.5 or a mean score≥2.5 is considered abnormal, with higher scores indicating a greater level of symptom severity.

The ETDQ-7 is a valid measure [11] and has been translated into German [12], Dutch [13], and Japanese [14]. The original English ETDQ-7 was translated into Arabic by 2 independent native Arabic doctors with excellent knowledge of the English language. The Arabic version was then translated again into English by 2 independent native English doctors with excellent knowledge of Arabic; the authors compared the back-translated version from Arabic to English with the original English version, and all differences were reconciled. This process was followed to make all aspects abundantly clear to readers.

To avoid recall bias, the participants were instructed to answer the questions of the ETDQ-7 based on the symptoms they had experienced in the past month [11]. The ETDQ-7 is a self-administered survey; yet, when needed, children received help from their parents to clarify and answer the questionnaire.

### Statistical Analysis

The data were organized in an Excel sheet and transferred to Statistical Package for Social Sciences (SPSS) software (version 24; IBM Corp.) for further analysis. Categorical variables were described using a frequency table, whereas continuous variables were described using the mean and SD. The data were then processed for determining statistical significance using the Chi-square test. For all statistical tests, *P* values<.05 were considered significant.

## Results

A total of 2372 participants agreed to contribute to our study, resulting in a response rate of 78.00% (2372/3041). The study population comprised 64.71% (1534/2732) female and 35.28% (837/2732) male participants, with a mean age of 31.31 (SD 11.85) years. The largest age group comprised 45.06% (1070/2372) of our total sample and included participants aged 19-29 years. Data on the past medical history of our sample showed that only 3.37% (80/2372) and 5.52% (130/2372) of the participants had been diagnosed with ETD and hearing loss, respectively. Among the total sample, 37.48% (889/2372) reported a positive family history of hearing loss. Other demographic characteristics of the population are presented in Table 1.

The results of our analysis of the participants’ responses to the ETDQ-7 demonstrated that the overall prevalence of ETD in our sample was 42.49% (1008/2372). The fourth question—regarding “the presence of ear symptoms when having a cold or sinusitis”—recorded the highest mean score of the entire questionnaire. The mean scores of all the questions in the ETDQ-7 are shown in Table 1.

**Table 1:** Demographic characteristics of the sample and mean results of the ETDQ-7 (N=2372).

Variables		Values		
Age, mean (SD)		31.31 (11.85)		
**Age groups (years), n (%)**				
	≤18	181 (7.63)		
	19-29	1069 (45.06)		
	30-39	486 (20.48)		
	≥40	636 (26.81)		
**Sex, n (%)**				
	Male	837 (35.28)		
	Female	1535 (64.71)		
**Nationality, n (%)**				
	Saudi	2137 (90.09)		
	Non-Saudi	235 (9.90)		
**Diagnosed with ETD^a^, n (%)**				
	Yes	80 (3.37)		
	No	2292 (96.62)		
**Diagnosed with hearing loss, n (%)**				
	Yes	131 (5.52)		
	No	2241 (94.47)		
**Family history of hearing loss, n (%)**				
	Yes	889 (37.47)		
	No	1483 (62.52)		
**Smoking, n (%)**				
	Yes	332 (13.99)		
	No	2040 (86.00)		
Overall prevalence of ETD according to the ETDQ-7^b^, n (%)		1008 (42.49)		
**Questions, mean (SD)**				
	1- Pressure in the ears?	1.961 (1.44)		
	2- Pain in the ears?	2.046 (1.47)		
	3- A feeling that your ears are clogged or ‘under water’?	2.280 (1.68)		
	4- Ear symptoms when you have a cold or sinusitis?	2.417 (1.81)		
	5- Crackling or popping sounds in the ears?	1.972 (1.59)		
	6- Ringing in the ears?	2.066 (1. 62)		
	7- A feeling that your hearing is muffled?	2.172 (1.72)		

^a^ETD: Eustachian tube dysfunction.

^b^ETDQ-7: 7-question Eustachian tube dysfunction questionnaire.

Upon comparison of the prevalence of ETD on the basis of the demographic and clinical features of our sample, the condition was found to be most prevalent among participants aged 18 years or younger (45.3%), and its prevalence was similar in the groups aged 19-29 and 30-39 years (41.8% and 41.2%, respectively). In this study sample, ETD was more prevalent among females compared to males and among Saudi participants compared to non-Saudi participants. The prevalence of ETD was significantly higher among participants who had reported a previous diagnosis of ETD and hearing loss (1897/2372, 80% and 1902/2372, 80.2%, respectively; *P*<.001) than among participants with no history of such diagnoses. Additionally, participants with a family history of hearing loss had a significantly higher prevalence of ETD (1136/2372, 47.9; *P*<.001) than those with a negative family history. Details of the prevalence of ETD in the sample are presented in Table 2.

Multivariate regression revealed that a diagnosis of hearing loss, family history of hearing loss, diagnosis of ETD, gender, and smoking were associated with the prevalence of ETD. These data are presented in Table 3.

**Table 2:** Prevalence of ETD according to the ETDQ-7 (N=2372).

Variables		ETD^a^	*P* value^b^
**Age groups (years), n (%)**			.65
	≤18	82 (45.30)	
	19-29	447 (41.81)	
	30-39	200 (41.23)	
	≥40	279 (43.92)	
**Sex, n(%)**			.01
	Male	326 (38.91)	
	Female	682 (44.39)	
**Nationality, n (%)**			.61
	Saudi	904 (42.30)	
	Non-Saudi	104 (44.34)	
**Diagnosed with ETD,** **n (%)**			<.001
	Yes	64 (80.00)	
	No	944 (41.23)	
**Diagnosed with hearing loss,** **n (%)**			<.001
	Yes	105 (80.21)	
	No	903 (40.33)	
**Family history of hearing loss,** **n (%)**			<.001
	Yes	426 (47.92)	
	No	582 (39.22)	
**Smoking,** **n (%)**			.17
	Yes	153 (46.09)	
	No	855 (41.91)	

^a^ETD: Eustachian tube dysfunction.

^b^*P* values were derived via the Chi-square test.

**Table 3:** Prevalence of ETD according to the ETDQ-7, based on multivariate analyses (N=2372).

Variables	R	Confidence interval	*P* value
Diagnosed with hearing loss	0.349	0.263 to 0.436	<.001
Diagnosed with ETD^a^	0.308	0.199 to 0.417	<.001
Family history of hearing loss	0.063	0.023 to 0.103	.002
Gender	–0.073	–0.117 to –0.030	.001
Smoking	0.064	–0.004 to 0.124	.04

^a^ETD: Eustachian tube dysfunction.

## Discussion

The aim of this study was to estimate the prevalence of ETD among the public in Saudi Arabia—which, to our knowledge, has not been measured before—using the ETDQ-7 instrument. ETD negatively affects the outcomes of middle ear surgery and is highly associated with OME and cholesteatoma [15]. Multiple studies have shown that patients with other diseases such as temporomandibular joint dysfunctions also have tinnitus, vertigo, hypersensitivity to sounds, and hearing loss, but no recent studies have focused on using a specific tool to assess the general population [16-18]. Knowledge of the prevalence and risk factors of ETD will help improve the treatment options for patients and provide a better understanding of the condition with regard to a particular population. The overall prevalence of ETD in our sample was 42.49% (1008/2372), with the presence of “ear symptoms when having a cold or sinusitis” reported most frequently. Although this prevalence rate can be considered high, it is difficult to make a definitive judgement in this regard since there are no recent studies on the prevalence of ETD among general populations. Moreover, the ETDQ-7 has a score range of 7-49, and since any score above 14.5 was considered abnormal, the narrow range of scores might have led to a high number of diagnosed cases of ETD. Note that the estimated prevalence of ETD in the UK population is 0.9%, which was described by that study’s authors as a “common” condition [4].

In this study, a family history of hearing loss was significantly correlated with the presence of ETD (*P*<.001). Thus, family history played a major role in ETD prevalence in our study. Similarly, family history of OM has previously been suggested as a genetic factor for the development of OM [19-21]. We believe that the same factors might have contributed to the high prevalence of ETD in our study. Moreover, since families live together and share the same lifestyle habits, they are equally vulnerable to the same environmental risk factors of hearing loss.

Among participants below the age of 18 years, 45% (1067/2372) were diagnosed with ETD, making them the age group with the highest prevalence of ETD in this study. This finding is similar to that of a German study, which found that least 40% of all German children are affected by ETD [5]. The other age groups in our study exhibited similar prevalence rates of ETD. This might be partly attributed to the fact that the city of Jeddah is very crowded. It is the second largest city in Saudi Arabia, with an estimated population of around 4.03 million [22]. The city has significant industrial capabilities and is highly developed. The high population density and industrial environment of the city might serve as a major risk factor for ETD.

An unexpected finding was that females were at a greater risk of developing ETD than males (*P*=.01). A previous large-scale study reported that women above the age of 20 years are at a greater risk of developing ETD/OME/tympanic membrane retraction than men in the same age group [8]. The social lifestyles of men and women are different in Saudi Arabia, which might lead to the differences in the risk factors between the two sexes. Currently, we are unable to explain the reason behind this finding in our study, and thus, further research on the differences in lifestyle-based risk factors between the two sexes is needed.

Multiple recent diagnostic evaluations have helped improve the understanding of the human anatomy; examples include the dynamic functions of the eustachian tube orifice via simple nasal endoscopy and other research-related diagnostic tests such as sonotubometry, tubomanometry, the pressure chamber test, and inflation deflation test [23-30]. Bearing this in mind, ETD is diagnosed when symptoms of pressure disequilibrium exist in either ear, particularly with symptoms of aural fullness, popping, or discomfort/pain [31]. To our knowledge, no previous study has used the ETDQ-7 to measure the prevalence of ETD; however, since this study is the first to do so, our findings, which show a high prevalence of ETD (up to 40%) in the community, might indicate an overdiagnosis. Although the questionnaire was validated, it has not been previously used in a study following a methodology similar to ours; moreover, the authors who validated the questionnaire mentioned that their work was limited by the small sample size [11]. Additionally, one of the questions in the ETDQ-7 asked if the individual experiences ear symptoms when they have a cold or sinusitis, to which many of the participants gave a score of 7. This question might have misled the participants and thus affected the prevalence rates in our study, suggesting that this question might need to be more specific. Thus, if the ETDQ-7 is to be used as a tool to assess the prevalence of ETD in a clinical sitting, the abovementioned question might affect its overall sensitivity considerably; indeed, this observation was also made by similar studies [32,33].

A major limitation of this research related to our inability to include people of all socioeconomic sections of society in Jeddah or ensure that our study covered all residential regions of the city. Future studies should consider different environmental risk factors when collecting data and attempt to identify an association between hearing loss and ETD. They should also evaluate the prevalence of ETD in all regions and neighborhoods of the city and consider the extent of noise exposure in each region. Another suggestion for future investigations would be to analyze the prevalence of ETD among vulnerable population groups such as those exposed to occupational and environmental risk factors. Our study design can be applied to larger studies on a national or an international scale, which will help compare the prevalence of ETD among different cultures and identify more specific risk factors for the condition. Given the high prevalence of ETD in our study, more research and awareness on ETD in the community are needed.

In conclusion, this study aimed to determine the prevalence of ETD among the public in Saudi Arabia by using the ETDQ-7. Our results showed that ETD is a common disease in Jeddah. The prevalence of ETD was significantly higher among participants who had reported a previous diagnosis of ETD and hearing loss. Therefore, this condition needs more attention, awareness, and research (particularly the use of advanced statistical analysis). In addition, since smoking has a significant impact on ETD and tends to cluster in families, the potential role of smoking in family history of ETD should be examined.

## Abbreviations

ETD: Eustachian tube dysfunction
ETDQ-7: 7-item Eustachian Tube Dysfunction Questionnaire
OM: Otitis media
OME: Otitis media with effusion
